# Early Numeracy and Literacy Skills Among Monolingual and Bilingual Kindergarten Children

**DOI:** 10.3389/fpsyg.2021.732569

**Published:** 2021-12-01

**Authors:** Liat Bar, Shelley Shaul

**Affiliations:** Department of Learning Disabilities, Edmond J. Safra Brain Research Center for the Studies of Learning Disabilities, University of Haifa, Haifa, Israel

**Keywords:** early literacy, early numeracy, kindergarten children, monolingualism, bilingualism

## Abstract

Early numeracy and literacy skills are all the knowledge that children acquire spontaneously and independently before entering school and beginning formal learning. This knowledge is essential and forms the basis for the acquisition of reading and arithmetic in school. A bilingual child is a child who is fluent in two languages, as opposed to a monolingual child who is exposed to only one language. Bilingualism has been found to affect verbal and mathematical abilities in children, but only a few studies have focused on the early numeracy and literacy skills of preschoolers. This study examined the connection between early numeracy and literacy skills and among monolingual children as compared to bilingual children in preschool. Three hundred and two children aged 5–6years old were recruited from 74 kindergartens. Participants were divided into two groups: 151 monolingual children who spoke and were exposed to only one language (Hebrew) and 151 bilingual children who spoke and were exposed to two languages (the bilingual children spoke different languages). Monolingual children performed better than the bilingual children in most of the literacy tasks, except for phonological awareness, in which no differences were found between the groups. In addition, in the early numeracy tasks, a difference was found only in the task, which included linguistic knowledge, number knowledge, and counting tasks, in which the monolingual children performed better. Furthermore, stronger correlations were found between the early numeracy and literacy skills among the monolingual group compared to the bilingual group. The study findings stress the importance of strengthening linguistic abilities, such as vocabulary expansion in kindergarten among populations in which more than one language is spoken. Supporting these abilities can reduce the gap between bilingual children and their monolingual classmates before entering school.

## Introduction

### Bilingualism

Most children around the world are exposed to more than one language. Bilingualism or multilingualism develops from learning two languages simultaneously or from initial learning of one language to which another language is added (McCardle and Hoff, 2006). Bilinguals are a heterogeneous group. There are many ways to acquire two languages: different contexts, different ages, simultaneous acquisition of two languages or in sequence, and different language pairs. It has been found that when children are exposed to two languages from infancy (early bilingualism), languages are generally better mastered than with late bilingual exposure (Kuhl and Ramirez, 2016). It is important to carefully characterize the language learning of infants and children in order to clearly understand and describe the differences in bilingual development. Identifying a profile of bilinguals that is different from monolinguals may help in tailoring their learning to help them gain needed skills, such as developing literacy in both languages (McCardle and Hoff, 2006).

The exposure of children from an early age to two languages at the same time poses challenges in language acquisition. Children are exposed to these languages from different sources (e.g., parents, other family members, friends, and the environment; Fennell et al., 2007). Studies show that acquiring and maintaining proficiency in two languages may reduce proficiency in the dominant language, whether it is the first language acquired or the second language (Misdraji-Hammond et al., 2014). Bilinguals who speak two languages sometimes must suppress one of their languages, while speaking a second language (Volmer et al., 2018).

Despite these challenges, there are also benefits of acquiring a second language. Studies have found that bilingual children have better abilities in executive functions and meta-linguistic skills (Adesope et al., 2010; Blom et al., 2014). Bilingual children have been found to have better selective attention and cognitive flexibility (Adi-Japha et al., 2010) during language use due to extensive practice of two languages. Because words in both languages are activated when one is used, bilinguals become accustomed to focusing their attention on relevant information (Bialystok, 2001). One example of this is the Stroop task, in which bilingual children performed significantly better (Poulin-Dubois et al., 2011); it should be noted that not all studies have found these advantages (for review see: Giovannoli et al., 2020).

In addition to the cognitive benefits of executive functions, there are also linguistic benefits for the bilingual group. Early studies have shown that bilingual children performed better than monolingual children on a variety of meta-linguistic awareness tests (Ben-Zeev, 1977; Bialystok, 1987). In a study that examined learning new words, which were not phonologically similar to any of the languages of the participants, an advantage in acquisition of new words was found in favor of the bilinguals (Kaushanskaya and Marian, 2009). The acquisition of languages begins at a young age, and spoken language forms the basis for the acquisition of the early literacy skills. Furthermore, bilingualism has been found to affect verbal and mathematical abilities in children; therefore, the current study will examine both of these domains.

### The Connection Between Linguistic and Numerical Abilities

Mathematical skills are closely related to language skills, which help children learn mathematics through the expression of mathematical thinking and understanding of mathematical concepts (Méndez et al., 2018). The development of the early knowledge of numbers is influenced by several non-mathematical factors, particularly by language skills (Purpura and Reid, 2016). It is suggested that simple numerical processing (calculation) depends on language, and therefore, early mathematical abilities are directly affected by language skills (Méndez et al., 2018).

Linguistic knowledge, such as general vocabulary and phonological awareness, has been found to be the most consistent and powerful predictor of early numerical abilities (Purpura and Reid, 2016). In everyday language learning, children use their knowledge of a word to learn new meanings and ideas, and a strong vocabulary may help them learn new numerical concepts in the early stages of numeracy learning (Méndez et al., 2018).

The strong connection between linguistic and numerical abilities is expressed at an early stage in children’s academic development. Children who have difficulty in both areas tend to encounter significant and ongoing challenges compared to those who have difficulty in one ability only. A study found that the link between language and mathematics was stronger among native speakers than among second language speakers (Peng et al., 2020). Another study, which examined the relationship between numeracy tasks and early verbal tasks among monolingual and bilingual children in kindergarten, found that the processing of letter and number symbols shared a common cognitive component independent of specific knowledge in literacy or numeracy (Bonifacci et al., 2016).

Although a consistent relationship between general language skills and mathematical abilities has been found from early childhood, improving general language does not always lead to a positive effect on mathematical ability, which may lead to the conclusion that the relationship between these two abilities is more complex (Purpura and Reid, 2016). Therefore, the aim of this study was to examine the relationship between these two skills in depth and to investigate whether these relationships are the same among monolingual children compared to bilingual children and whether bilingualism affects the connection between language and mathematics.

### Early Literacy

Early childhood is a critical stage for the development of the early literacy skills. Studies have found that the level and quality of language that a child experiences during early childhood has a significant impact on school readiness and academic performance. Early literacy is acquired spontaneously from exposure to language in the child’s environment and from learning activities such as book reading (Li et al., 2014). Sénéchal and LeFevre (2014) found that home literacy environment (HLE) has a significant role in developing early literacy abilities and in promoting reading. These findings accentuate HLE as an important factor in the development of reading abilities and linguistic skills. Moreover, it appears that forming a “language-rich environment” advances vocabulary and linguistic skills and is an important factor in promoting the early literacy abilities of preschoolers.

Exposure to preschool literacy knowledge is an important basis for developing later academic skills (Duncan et al., 2007). Early literacy is also characterized by an interest in written things, including an awareness of letters, words, sounds, and forms that appear in language. This interest is manifested in the preoccupation with books and texts and initial attempts at reading and writing, not necessarily in accordance with the language conventions accepted by adults (Whitehurst and Lonigan, 1998). When children are exposed to new situations and new information, opportunities for cognitive and social development are created, including the development of literacy (Lukie et al., 2014).

Early literacy encompasses several areas of knowledge: phonological awareness, orthographic knowledge, morphological awareness, and vocabulary.

*Phonological awareness* refers to the ability to recognize spoken words and break them down into their sounds, such as phonemes and syllables (Abu-Rabia et al., 2013), and the understanding that a word is composed of sounds and that the letters represent different sounds (Ehri, 2005). This awareness of the connection between phonemes and the sounds of the letters is a basic ability, which develops reading and writing and is considered to predict reading in alphabetic languages, not only in the mother tongue but also in a second language (Abu-Rabia et al., 2013). Though phonological awareness underlies reading development, it does not depend on the specific language spoken by bilingual children (Leikin et al., 2010).

*Orthographic knowledge (print conventions)* refers to knowledge of the written structure of a particular language and consists of orthographic (visual) symbols in written words that help to identify words (Abu-Rabia et al., 2013). The ability to recognize letters based on visual aspects affects the development of early literacy, and exposure to words is enough to bring about orthographic learning (Schmitterer and Schroeder, 2018). It has been found that children learn to identify visual features of letters from the age of three, and when asked to write at this age, they usually do not use drawings as a form of writing (Gombert and Fayol, 1992). One of the hallmarks of reading acquisition among novice readers is familiarity with letter names. Knowing the names of letters often helps in accessing their sounds. To create effective second language word processing, mastery of orthographic knowledge must reach the level of first language automaticity (Abu-Rabia et al., 2013).

*Morphological awareness* refers to the knowledge needed in order to recognize a word, understand the forms, which create it, and produce morphologically complex words. Morphology is essential for the acquisition of reading skills because it contributes to the expansion of vocabulary and as vocabulary grows knowledge about the internal structure of words increases (Bar-On and Ravid, 2011). Studies have shown that morphological awareness affects reading comprehension and acquisition of spelling skills (Abu-Rabia et al., 2013).

*Vocabulary and morphological knowledge* refers to knowledge about words and word parts. Vocabulary is divided into two parts: depth of knowledge and breadth of knowledge. Breadth of knowledge refers to the number of words learned, and depth of knowledge refers to the quantity and quality of a person’s knowledge of individual words. Knowing a word includes more than its definition. Most of the learning of words occurs when the word is encountered several times, and for this to happen, the student must be exposed to large amounts of input. Second language learners usually need to learn at a faster rate than was necessary during the acquisition of their first language, because the second language is usually taught and not acquired gradually in a developmental manner through exposure (Nagy, 1995).

Studies conducted among school-aged children suggest that vocabulary skills are a significant predictor of reading and academic achievement in monolingual children (Lee, 2011). It was also found that bilingual children’s vocabulary in each of their languages is smaller than the vocabulary of monolingual children (De Houwer, 2007; Oller et al., 2007). Vocabulary is known to be significant for the development of mathematical skills as well, such as understanding mathematical concepts (Purpura et al., 2011), and it predicts computational skills at a later age (Purpura and Ganley, 2014).

The development of young children’s literacy skills includes learning the system of reading and writing, as well as the components of oral language – the phonological, morphological, syntactic, and lexical aspects that characterize texts of that language. Kindergarten children with developed literacy skills have been found to experience success in acquiring the alphabetic code and becoming skilled readers at the beginning of formal reading instruction. In contrast, children with low literacy skills will most likely face difficulties during the acquisition of reading in first grade (Arafat et al., 2017).

#### Bilingualism and Verbal Abilities

Bilingualism affects verbal abilities in both the first and second language. It has been found that bilingual children with reading difficulties in their mother tongue also showed difficulty in the second language. The connection between reading and writing skills in the first language and the second language is explained by oral language abilities, such as phonological, orthographic, and morphological awareness, which form the base of reading. If linguistic skills are strong in the first language, we observe the same level in the second language (Abu-Rabia et al., 2013).

Bilingual children often have difficulty acquiring early literacy skills compared to their monolingual peers (Hur et al., 2020). They often show lower achievement in school compared to their monolingual peers, which may result from the fact that the instruction at school occurs in a different language to which they speak at home. A study that examined early literacy skills among monolingual (English) and bilingual children in preschool found an improvement in early English literacy skills among bilinguals who took part in a language intervention program, which focused on vocabulary enrichment. The researchers conclude that it is important to address the unique characteristics of each child (e.g., language proficiency and language exposure), in order to promote each child’s ability and strengthen their weakness (Hur et al., 2020). The literature also shows that the phonological processing skill that underlies reading development does not depend on the specific language that bilingual children speak (Leikin et al., 2010).

In addition, mastery of more than one language has been found to affect naming, which is related to language skills (Misdraji-Hammond et al., 2014). Naming and reading are interrelated in that they both depend, among other things, on the rapid execution of basic processes (Cui et al., 2017). Studies have found that bilingualism affects naming skill due to the competition between the retrieval of the word in both languages, and therefore, the naming skill of monolinguals is better than that of bilinguals, which are slower in their naming speed (Misdraji-Hammond et al., 2014).

Another study, which examined the differences in verbal abilities of English-speaking monolingual children compared to English-Spanish bilingual children, found similar performance in basic reading tasks, but there was a significant difference in the vocabulary task. The bilingual children knew fewer words in each one of the languages as compared to the monolingual children (Oller et al., 2007).

The studies above demonstrate that bilingualism can influence the acquisition of children’s language skills, which later affect literacy abilities. It seems that bilingual children have longer trajectories for language acquisition and development, which may affect the timing of school-based learning of skills like literacy. The question is whether this tendency will be found also in the different aspects of early literacy skills of bilingual children.

### Early Numeracy

The development of early numeracy in children occurs during the kindergarten years, before the beginning of the formal education (Méndez et al., 2018). The development of these early quantitative abilities is a complex and ongoing process; researchers noted three routes by which children typically acquire numerical abilities: linguistic knowledge, executive functions, and numeracy knowledge (Starkey et al., 2004; Sarama and Clements, 2009; Purpura and Reid, 2016). Early numeracy skills include counting, identification of quantities, and the initial ability of addition and subtraction. These abilities develop gradually over time (Méndez et al., 2018). Early numeracy skills in kindergarten predict mathematical achievement years later: in elementary school, middle school, and even high school (Duncan et al., 2007). The process of development of numerical abilities does not occur independently (Purpura and Reid, 2016). Like early literacy, it is influenced by the environment and learned from exposure and various quantitative activities at home and in the environment.

Early numeracy includes several areas of knowledge: number knowledge, comparison of quantities, simple calculation, and verbal problem-solving.

*Number Knowledge.* Early number knowledge usually begins when young children learn to recite a list of numbers while counting. Learning to how to count and the correct order of numbers helps to build the understanding that the smaller numbers come before the bigger numbers. The knowledge that the order of numbers represent their amount forms the basis for symbolic representation of quantities at a later stage (Méndez et al., 2018). Number knowledge is one of the strongest predictors of mathematical achievement at school (Viesel-Nordmeyer et al., 2019). Findings from a meta-analysis suggested that early mathematical concepts, such as knowledge of numbers and their order were strong predictors of late mathematical learning (Duncan et al., 2007).

*Comparison of Quantities.* Understanding mathematical language is probably also related to comparing groups, in which children look at groups of dots and determine which group has the larger or smaller amount of dots, and digit comparison, in which children identify which numbers are larger or smaller (Hornburg et al., 2018).

*Simple Calculation.* This knowledge is built from the ability to disassemble and assemble quantities, as well as an early understanding of the concepts of addition and subtraction (Clements and Sarama, 2007). It has been found that infants are not only sensitive to numbers, but they can also even perform calculation operations (Mix et al., 1997).

*Verbal Problem-Solving.* This task requires complex processes above computational skills, such as reading comprehension, using linguistic information, identifying relevant information, and creating an appropriate arithmetic exercise (Swanson et al., 2021). According to mathematical development, number-related skills are necessary for solving problems that are more complex. Without concepts, children will have difficulty in more complex understanding of mathematics, for example, in applying numerical knowledge to solving verbally presented word problems (Viesel-Nordmeyer et al., 2019).

Of particular importance is the development of a variety of mathematical skills in kindergarten, such as understanding cardinality, counting, size comparison, and basic arithmetic calculation. Recent studies suggest an association between low early numeracy skills and mathematical difficulties that persist even during schooling (Viesel-Nordmeyer et al., 2019). Several studies found that number recognition abilities, distinguishing between quantities, and identifying missing numbers in certain sequences predict mathematical abilities at the end of first grade (Clarke and Shinn, 2004; Chard et al., 2005). Another study found a strong, significant, and ongoing predictive relationship of early numeracy skills from the kindergarten period to later math’s skills the third grade. These findings show the importance of early mathematical abilities as a basis for later success in elementary school mathematics (Jordan et al., 2009).

#### Bilingualism and Mathematical Abilities

The relationship of bilingualism to verbal ability has been investigated over the years, but the effect of bilingualism on mathematical abilities has been less examined. The relationship between linguistic and numeric skills has been established by many studies (e.g., Van Rinsveld et al., 2016). In a study, which examined the learning of multiplication facts among bilingual children, the children were tested in both languages. They performed the task better when the language of instruction matched the language of the test; hence, the language of instruction of mathematics affects situations where knowledge needs to be applied in a new context, as is often required in the classroom (Volmer et al., 2018).

Another study, which examined the differences in numerical abilities between bilingual and monolingual preschool children, found no significant differences between the two groups (Iglesias, 2012). In contrast, another study, which followed bilinguals from kindergarten to elementary school years, found benefits for bilinguals in mathematical abilities from basic skills in kindergarten to elementary school mathematical knowledge (Hartanto et al., 2018). Furthermore, a study, which examined the early numeracy abilities of bilingual and monolingual preschool children found differences in favor of the monolingual children in the numeracy tasks with a verbal component, such as number knowledge, but no differences in the tasks with non-verbal components, such as comparing quantities (Bonifacci et al., 2016).

It can be concluded that bilingualism can also affect numeracy skills, ranging from mastering early basic skills in preschool to developing these skills in formal school learning. There are not many studies that have examined the differences in mathematical abilities between bilingual and monolingual children in kindergarten except for the few, which are mentioned above, mathematical skills, have been explored mainly among older children. However, at the preschool level, different findings have been observed regarding the differences between the groups as presented above. Further research is needed to examine this topic and investigate whether the differences between bilingual and monolingual children appear from an early age and in which skills.

### The Current Research

A single study was found that examined the relationship between verbal abilities and early numeracy abilities between monolingual (Italian) and bilingual children in early childhood. The study examined the differences in numeracy and linguistic abilities between the two groups, as well as the linguistic predictors for early numeracy skills. This study found that the monolingual children performed better than the bilingual children did in most of the early literacy skills as well as in numeracy tasks with a verbal component only. In addition, different predictors were found for the early numeracy skills, while letter knowledge was found to be a significant predictor of numeracy tasks with a verbal component in both groups of children, phonological awareness was a predictor of numerical ability only among the monolingual children (Bonifacci et al., 2016).

Studies further suggest that the most powerful predictor of later mathematical performance is prior mathematical knowledge. Nevertheless, linguistic abilities also affect the learning of mathematics. The linguistic abilities found to influence math performance include spoken language skills, such as vocabulary and verbal comprehension, while phonological processing has been linked to the development of mathematical skills (Foster et al., 2015). Therefore, an examination of the relationship between these two areas is extremely important.

In addition, bilingualism has been found to affect verbal and mathematical abilities in children, but the findings are not uniform, and different studies have found different associations between these abilities. Most of the studies have investigated older children, and very few studies have examined both linguistic and numeric abilities in parallel on the same population. Moreover, due to the effect of bilingualism on the development pace of different linguistic abilities, which are related to different mathematical abilities, the question that arises is whether there are differences between monolingual and bilingual children in performing different skills of early literacy and numeracy in kindergarten. In addition to whether there are differences in the relationship between linguistic and numeric abilities in bilingual children as compared to monolingual children in kindergarten.

#### Research Questions

1. Is there a difference between monolingual and bilingual children in the different early literacy skills (phonology, print conventions, morphology, and oral language)?

It is hypothesized that differences will be found between the different groups, and bilingual children will perform the tasks less well than monolingual children will; the differences will be stronger in orthographic and language knowledge due to the different exposure to the two languages (Prevoo et al., 2016). However, difference in the phonological awareness task will be smaller since this task has been found to be independent of the specific language (Leikin et al., 2010).

2. Is there a difference between monolingual and bilingual children in the different early numeracy skills (counting, comparison, stock, and simple calculations)?

Although some studies have found that bilinguals were better than monolinguals in the numeracy field (Hartanto et al., 2018), most of the literature suggests that the monolinguals perform better in different numeric tasks, especially the tasks, which involve linguistic abilities, such as counting and verbal problems. It is hypothesized that differences will be found between the two groups and that the difference will be larger between the groups on the language-based tasks as compared to tasks based solely on mathematical knowledge (Iglesias, 2012).

3. What is the association between early literacy and numeracy skills among monolingual children and is it different among bilingual children? It is expected, based on previous studies that a link will be found between linguistic and numeric abilities among monolingual children and among bilinguals, although it is estimated that the connection will be weaker among bilinguals due to the lower verbal abilities but not mathematical.

## Materials and Methods

### Participants

The study examined 302 kindergarteners, 5–6years old, who were recruited from 74 different kindergartens in different areas. The children were from a variety of socioeconomic status backgrounds (low to high). SES was determined according to parent’s education and income as well as the neighborhood in which the kindergarten was situated. The distribution of the SES’s was similar in both groups: monolingual children 14.3% low SES, 64.6% medium SES, and 21.1% high SES. The bilingual group: 11.4% low SES, 56.5% medium SES, and 32.1% high SES. It is important to note that there was no significant interaction between SES and the groups of children and SES effected both groups of children in the same way with high SES performing better than low SES in all early literacy and numeracy measures.

Participants were divided into two different groups: 151 monolingual children with an average age of 5years and 8months (68 boys and 83 and girls), who spoke only one language (Hebrew) and were not exposed to other languages at home, and 151 bilingual children with an average age of 5 and 9months (77 boys and 74 girls) who spoke Hebrew as well as one of the following languages: Russian, English, Spanish, Japanese, German, Arabic, Ukrainian, Hungarian, Portuguese, Romanian, Persian, Italian, Armenian, Amharic, Georgian, or French. The children in the bilingual group were all exposed to two languages but were fluent in the Hebrew language. All the children in both groups understood all the instructions of the different tasks and performed all the linguistic and numeric tasks in Hebrew. In addition, all the children were in a Hebrew-speaking kindergartens and communicated with the teachers and other children only in Hebrew.

Both monolingual and bilingual children were chosen from each kindergarten in order to neutralize the effect of the quality of the teaching as well as the socioeconomic status of the children. Data were collected after receiving a consent form signed by the parents of the children who participated in the study. All children were in regular education and did not have any developmental or neurological problems.

### Research Tool

Demographic questionnaire: All parents filled out a questionnaire regarding details about the place of birth, the language spoken at home, and the child’s mastery of the different languages in order to verify the child’s bilingualism.

#### Early Literacy Skills

1. Orthographic Knowledge (based on Schwartz, 2004, unpublished).
  - Letter naming. The child was asked to name 10 letters in the Hebrew language, which were presented to him or her. The total amount of letters named correctly was scored (*α*=0.87).
  - Letter identification. The child was asked to identify a specific letter from four letters, which were visually displayed to him or her. The total amount of letters recognized correctly was scored (*α*=0.82).
  - Orthographic identification of words (Shaul, 2015, unpublished). This task is based on a similar Dutch test (Van der Kooy-Hofland et al., 2012). A word is presented to the child orally, and he or she has to identify it from four printed words. The distractors from the target word differ by one letter, two letters, or all the letters. The final score consists of the sum of the points received from the 10 items displayed (*α*=0.75).
2. Phonological Awareness (Share and Gott, 2018, unpublished).
  - Isolation of opening syllable. The test included 12 items in which the child was asked to say a word and then isolate the opening syllable and say the single syllable. The total number of correct isolations was scored (*α*=0.84).
  - Isolation of a closing consonant. The test includes 12 items in which the child was asked to say a word and then isolate the closing consonant and say the constant. The total number of correct isolations was scored (*α*=0.81).
3. Linguistic Knowledge and Vocabulary (Share and Gott, 2018, unpublished).
  - Vocabulary. The picture-naming task is based on the vocabulary subtest from a language screening test for preschool Hebrew-speaking children. The test contained 14 colored pictures. The children were asked to name each picture out loud following the examiner’s instructions (e.g., “What is this?” and “What is he doing?”). The score was based on the total number of pictures named correctly (*α*=0.84).
  - Morpho-syntactic skills: nonwords derivation task (Shalev-Laifer and Share, 2016, unpublished). The test included 10 sentences that were presented orally by the examiner. Each sentence contains a novel verb (a combination of root and conjugation) which represents a nonsense word in the Hebrew language. The children were required to complete the sentences by modifying and producing the verb in the correct inflection and derivation according to the Hebrew morpho-syntactic structures (*α*=0.65).
  - Noun plural production. Children were shown colored pictures. One picture contained a singular count noun item, and the second contained four of that same item. The child was asked to produce the noun in plural. The total of correct answers was calculated (maximum=15; *α*=0.74).
  - Consequential adjective production. The test contained 10 items. Children were shown two colored pictures. While pointing to the first picture the examiner said a sentence with a target verb, for example: “They broke the window.” Then, the examiner pointed to the second picture (with a broken window) and asked the children to complete the sentence by deriving the consequential adjectives from the verb (*α*=0.74).
  - Consequential verb production. The test includes eight sentences read aloud by the examiner. The children were required to complete the sentence by deriving the consequential verb from a noun. The total of correct answers was calculated (*α*=0.74).

#### Early Numeracy Skills

Early numeracy skills tasks were built based on tests of Purpura et al. (2011, 2015, 2017).

1. Number Knowledge
  - Verbal counting (forward and backward). Measured by two subtests in which children were asked to count aloud forward from 1 to 20 and backward from 10 to 1 or 0. Each pair of consecutive number words correctly pronounced received one point up to the number correctly counted according to the sequence.
  - Number naming. Children were required to verbally name 13 Arabic numerals (from 0 to 12). The numbers were presented in random order. Each number named correctly received one point (*α*=0.89).
2. Quantities Comparison
  - Symbolic and non-symbolic magnitude comparison from the numeracy screener test (Nosworthy et al., 2013). In the symbolic magnitude comparison, the children were asked to decide which of the numbers was larger in each single-digit numerical pair. In the non-symbolic magnitude comparison, the children were required to recognize the larger magnitude of two arrays of dots without counting. A total number of the correct answer within a 1-min time limit was calculated for each subtest (*α*=0.95).
3. Simple Calculations
  - Basic arithmetic task with the numbers 1–5. The child was presented with 10 simple addition and subtraction exercises, five of each type, using numbers up to 5, and he or she was asked to solve them orally. (e.g., 2+1, 2+2, 4–1) Measured: The number of correct answers out of 10 (*α*=0.79).
4. Verbal Problems
  - Arithmetic story problems. The test consisted of four addition and subtraction word problems (with numbers between 1 and 5). Each item was read to the child, who was then asked to solve the problem by stating a number word verbally. One point was given for each correct answer (*α*=0.64).

### Procedure

Prior to the collection of the data, the required approvals were obtained from the Ministry of Education and the Ethics Committee of the university. In addition, consent forms were signed by the parents of the children examined. All the tests were administered to the participants individually during kindergarten class time but in a separate room, in two or three separate sessions of about 20min each.

## Results

### Preliminary Analysis

In order to reduce the number of variables, two principal component analyses with varimax rotation on the measures of early literacy and numeracy measures were conducted.

All literacy measures included in the factor analysis yielded three major factors: oral language knowledge accounted for 28.67% of the variance, phonological awareness accounted for 21.63%, and alphabetic and orthographic knowledge accounted for 20.67%. All these factors together explained 71% of the variance in early literacy. The loadings of the different tests on the factors are presented in Table 1.

**Table 1:** Loadings and factor division findings for the different literacy tests.

Name of the test	1 Orthographic ability	3 Linguistic abilities	3 Phonological awareness
Letter naming	0.90		
Letter identification	0.89		
Word identification	0.66		
Producing verbs		0.56	
Producing plural		0.82	
Vocabulary		0.81	
Producing adjectives		0.81	
Inflecting verbs		0.81	
Isolation of initial sound			0.87
Isolation of final sound			0.73

All math measures included in the factor analysis are reported in Table 2, the measures yielded three factors: number knowledge (Factor 1), comparison of quantities (Factor 2), and arithmetic operations (Factor 3). Factor 3 included both simple calculation and verbal problems, but these were not merged due to the different forms of presentations (verbal vs. numbers) and were divided into basic arithmetic calculations and verbal problems separately due to the linguistic factor.

**Table 2:** Loadings and factor division findings for the different numeracy tests.

	Component		
	Factor 1: Number knowledge	Factor 2: Quantity comparison	Factor 3: Arithmetic operations
Number naming	0.580		
Ascending counting	0.837		
Descending counting	0.811		
Arithmetic facts			0.683
Arithmetic story problems			0.866
Non-symbolic magnitude comparison		0.919	
Symbolic magnitude comparison		0.866	

The first factor, which included number naming and counting (forward and backward), accounted for 26.29% of the variance. The second factor, which included arithmetic facts, calculation, and arithmetic story problems, accounted for 23.60% of the variance. The third factor, which included quantity comparison (symbolic and non-symbolic) measures, accounted for 19.70% of the variance. All three factors together explained 69.6% of the variance. The loadings of the different tests on the factors are presented in Table 2.

Based on the factor analysis aggregated variables were computed for each factor, and the average was computed on the percent of the correct answers n each task in each factor.

### First Research Question

**Figure 1:**
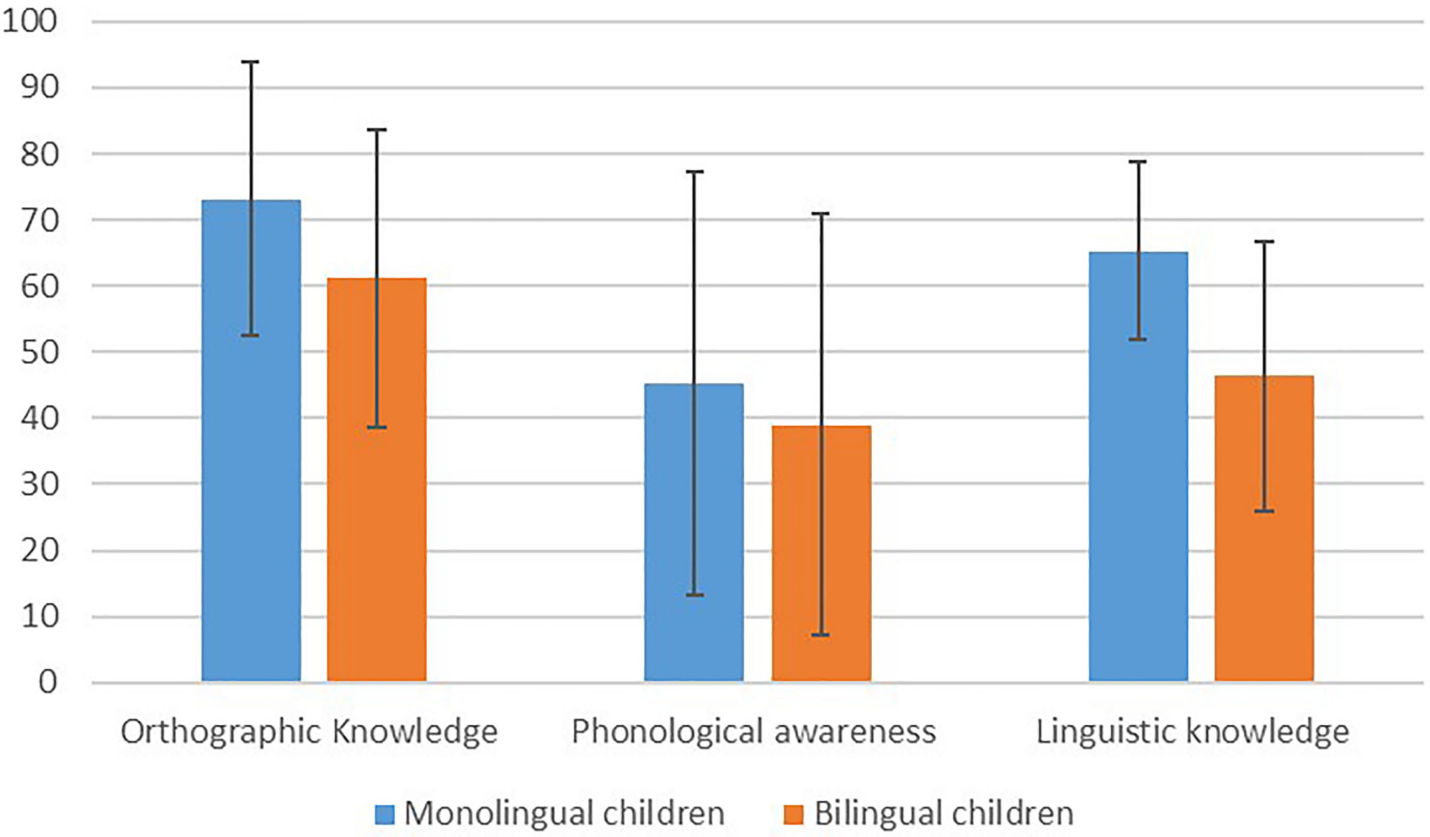
The performance of monolingual and bilingual children on the different early literacy factors.

The first research question examined whether there were differences in the various early literacy skills between monolingual and bilingual children. In order to answer this question, a MANOVA with SES as a controlled covariant was conducted for all the literacy measures. Table 3 and Figure 1 present the averages and SDs of the early literacy factors of each group of children.

**Table 3:** Means and SD of the different early literacy factors among the different groups of children.

	Monolingual children			Bilingual children		
	*N*	*M*	*SD*	*N*	*M*	*SD*
Orthographic Knowledge	141	73.15	20.61	139	61.14	22.55
Phonological awareness	136	45.32	32.09	129	39.04	31.84
Linguistic knowledge	141	65.26	13.44	135	46.38	20.39

A significant effect for group was found for the overall early literacy measures, *F*_(3,211)_=30.87, *p*<0.001. Significant differences were found between monolingual children and bilingual children in the measures of orthography, *F*_(1,211)_=21.49, *p*<0.05. The monolingual children performed better in orthographic knowledge (*M*=73.15; *SD*=20.61) than the bilingual children (*M*=61.14; *SD*=22.55). In addition, a significant effect for group was found for linguistic knowledge, *F*_(1,211)_=83.46, *p*<0.001. The same pattern was found in the linguistic knowledge of monolingual children with lower performance (*M*=65.26; *SD*=13.44) as compared to the bilingual children (*M*=46.38; *SD*=20.39). In the phonological awareness factor, no significant differences were found between the two groups of children.

### Second Research Question

**Figure 2:**
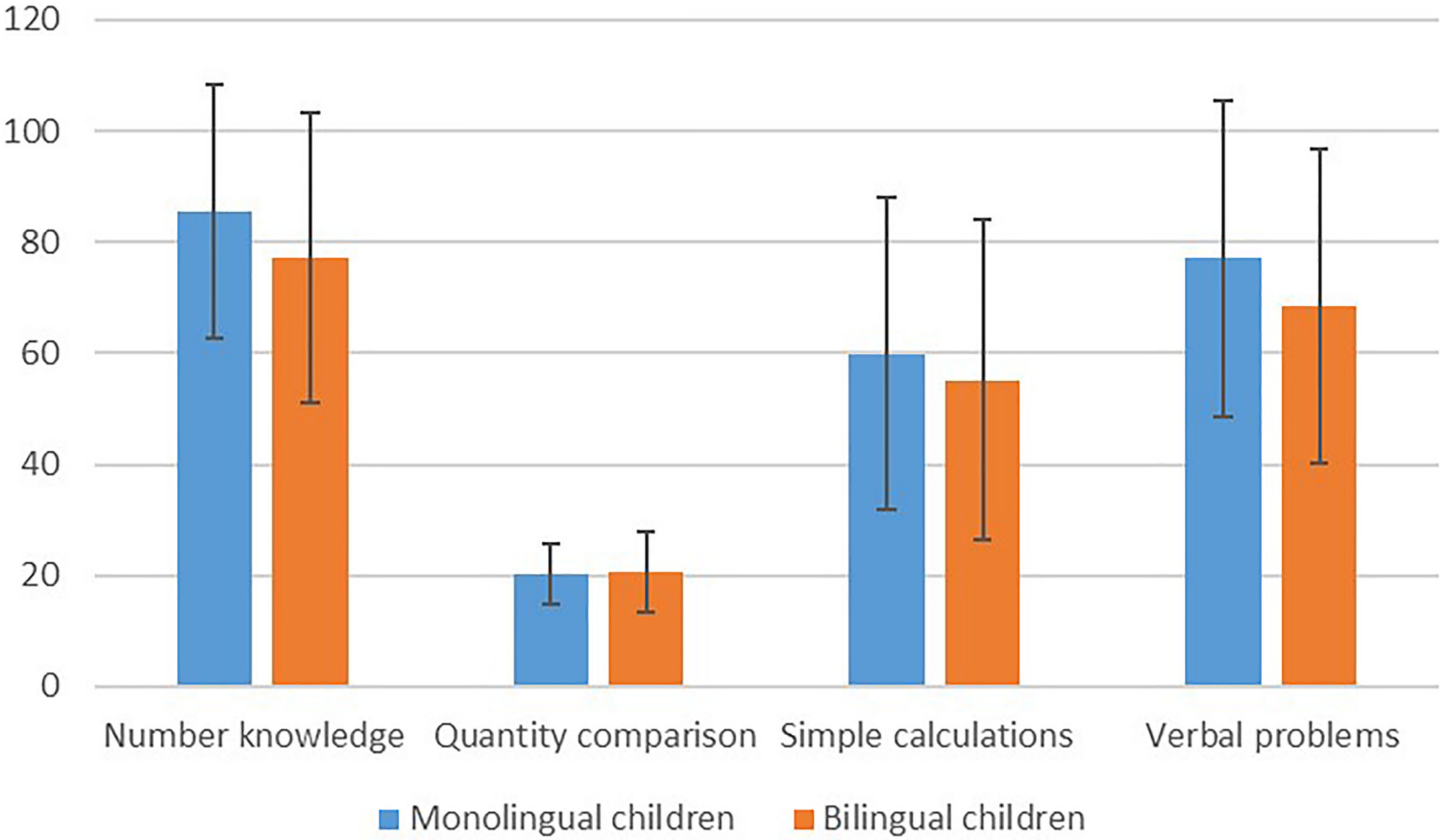
The performance of monolingual and bilingual children on the different early numeracy factors.

The second research question examined whether there were differences in different early numeracy skills between monolingual and bilingual children. In order to answer this question, a MANOVA with SES as a controlled covariant was conducted for all the numeracy measures. Table 4 and Figure 2 present the averages and SDs of the early numeracy factors of each group of children.

**Table 4:** Means and SD of the different early numeracy factors among the different groups of children.

	Monolingual children			Bilingual children		
	*N*	*M*	*SD*	*N*	*M*	*SD*
Number knowledge	145	85.49	22.79	143	77.13	25.95
Quantity comparison	148	20.22	5.47	145	20.68	7.13
Simple calculations	144	59.86	28.08	139	55.11	28.85
Verbal problems	145	77.03	28.51	144	68.40	28.36

A significant effect for group was found for the overall early numeracy measures, *F*_(4,222)_=2.97, *p*<0.05. Significant differences were found between monolingual children and bilingual children only in the number knowledge factor, *F*_(1,211)_=5.32, *p*<0.05, with the monolingual children performing better than the bilingual children (*M*=85.49; *SD*=22.79) as compared to the bilingual children (*M*=77.13; *SD*=25.95). In all other factors, no significant differences were found between the two groups.

### Third Research Question

The third research question examined the association between early literacy and numeracy skills among monolingual children and whether the connection is different among bilingual children. In order to examine this question, Pearson correlations were conducted between the various indices within each group. Table 5 shows the results of the correlations between each of the three linguistic factors and each of the four numeric factors measured, among the monolingual and bilingual children.

**Table 5:** Correlation between linguistic and numeric factors among monolingual and bilingual children.

Task	Monolingual children			Bilingual children		
	Orthographic Knowledge	Phonological awareness	Linguistic knowledge	Orthographic Knowledge	Phonological awareness	Linguistic knowledge
Number knowledge	^0.514^**^	0.455^**^	^0.566^**^	0.321^**^	0.331^**^	0.285^*^
Quantity comparison	0.138	0.141	0.149	0.213^*^	0.182^*^	0.103
Simple calculations	0.422^**^	0.492^**^	^0.476^**^	0.373^**^	0.372^**^	0.238^**^
Verbal problems	^0.422^**^	0.260^**^	^0.458^**^	0.164	0.176^*^	0.249^**^

** *p*<0.01;

* *p*<0.05.

^The correlations that were significantly higher among the monolingual as compared to the bilingual children are in bold.

Results revealed that among monolingual children, a significant association was found between most of the numeric tasks and the linguistic tasks, apart from the task of comparing quantities, which did not correlate with any of the linguistic tasks. Among the bilingual children, a significant correlation was found between number knowledge and simple calculations with all three linguistic tasks. The quantity comparison task correlated with the orthographic and phonological awareness tasks, but not with the linguistic knowledge and vocabulary tasks, and the verbal problems were related to phonological awareness and linguistic abilities but not to orthographic knowledge.

In order to compare the correlations between the monolingual and bilingual group, a Fisher analysis was conducted. It was found that the correlation between orthographic knowledge and number knowledge task was higher among the monolingual children (*Z*=1.95, *p*<0.05) as well as between the linguistic knowledge and number knowledge (*Z*=2.88, *p*<0.01). In addition, the correlation between linguistic knowledge and simple calculation was also higher among the monolingual group (*Z*=2.27, *p*<0.01). Finally, the correlation between orthographic knowledge and verbal problem slowing was higher among the monolingual children (*Z*=2.35, *p*<0.01) as well as between the linguistic knowledge and verbal problem-solving (*Z*=1.99, *p*<0.05). All the correlations are detailed in Table 5.

## Discussion

The current study examined whether there is a difference in the early literacy and numeracy abilities among monolingual children as compared to bilingual children, as well as examining the connections between these two abilities among the different groups of children. The findings are consistent with the research literature and lead to generalizations regarding all children who speak any two languages, who usually exhibit lower performance than monolingual children in linguistic and print dependent tasks.

### Differences in Early Literacy Abilities

The first research question examined whether there were differences in early literacy abilities between monolingual and bilingual children. Consistent with our hypothesis, significant differences were found in the measures of orthography and linguistic knowledge, in which the performance of monolingual children was better than the performance of bilingual children. These results support the findings in the literature that bilinguals are known to master one language more than the other and that similar proficiency in both languages is considered rare (Misdraji-Hammond et al., 2014). In addition, these children, who speak two languages, sometimes have to suppress one of their languages while speaking the other language (Volmer et al., 2018). This may explain the differences found between the groups in the literacy tasks.

It can be assumed that knowledge of the Hebrew language among bilingual children was lower, probably due to less exposure to Hebrew. Most of the children in the study came from homes where other languages are spoken and they were exposed to Hebrew mainly in kindergarten, so they had fewer opportunities to learn Hebrew vocabulary as compared to children who were exposed only to Hebrew. In addition, their print knowledge was less developed, since their print environment at home likely consisted of books in other languages.

Regarding phonological awareness, no differences were found between the two groups. Phonological abilities are based mainly on auditory ability, or sensitivity to the sounds of the language, which develops in the same way among all children whether or not they are bilingual. The literature provides support for the notion that the development of phonological processing – the ability to analyze and process auditory information – does not depend on the specific language to which you are exposed (Leikin et al., 2010). Hence, it can be concluded that when there is significant exposure to any oral language at home, no matter which one, then children can perform the phonological awareness task.

In conclusion, it can be seen that there are significant differences between monolingual children and bilingual children in most early linguistic literacy abilities, which form the basis for formal learning in school. In the phonological awareness task, one of the significant abilities predicting reading in school, no differences were found. In contrast, in the orthography and linguistic knowledge tasks, major differences were found. Similar findings were found in other studies in which bilingual children have lower early literacy skills than monolingual children (Hur et al., 2020). These findings suggest that some abilities probably develop in same way in all children (phonological awareness), whether bilingual or monolingual. In contrast, in orthographic knowledge and linguistic and vocabulary knowledge, significant differences were observed between the groups, in favor of the monolingual children. It is evident that these tasks are based on verbal abilities and require knowledge and mastery of a specific language in order to be performed optimally.

Therefore, in their work with bilingual children, educators should emphasize tasks that involve language knowledge, such as vocabulary expansion and orthographic tasks. It is possible that with the help of fieldwork following these findings, bilingual children will be able to bridge the gap with the monolingual group and even reach a level similar to theirs.

### Differences in Early Numeracy Abilities

The second research question examined whether there are differences in early numeracy abilities between monolingual children and bilingual children. The results of the study were in line with the previous research and found that only in the number knowledge task, which involves verbal and vocabulary abilities, is there a very clear difference between the two groups. That is, it is evident that main differences in verbal and numeracy abilities between the two groups of children were in naming and vocabulary. In contrast, in the three additional numeracy tasks – comparing quantities, solving simple calculations, and verbal problems – no differences were found between the two groups of children. It can be assumed that these three tasks are pure numeracy skills and are not based on language knowledge or that the language knowledge they require is less significant, at least at the level of kindergarten tasks, and therefore, no differences were observed.

With respect to the task of comparing quantities, since the literature shows that this task does not involve verbal abilities, we assumed that no difference would be found between the groups in performing the task, as found by Bonifacci et al. (2016). Comparing quantities of dots or numbers is a task that requires visual-spatial ability and relies on mechanisms of pure number processing, so if children understand the meaning of the number and their perception of quantity is intact, they can perform the task regardless of the language they speak.

According to the literature, there are also verbal aspects to solving simple sums through calculation (Harvey and Miller, 2017). Even so, in a study examining the differences between bilingual and monolingual kindergarten-age groups, no significant differences were found between the two, and numerical abilities were not found to be related to language abilities at this age (Iglesias, 2012). Hence, it seems that there are different approaches regarding language involvement. Solving simple sums in kindergarten may rely more on numeracy meaning or memory since from a young age there is exposure to and repetition of counting to 10 and rehearsing simple sums in the range of these numbers. It may be that differences will be found between the two groups when they are required to calculate more complex exercises later in their development.

Regarding the task of solving verbal problems, we expected to find differences between the two groups, since this task involves language knowledge and vocabulary. No differences were found between the groups in this task, and this may be because the numeracy abilities required to perform this type of task in preschoolers are more significant than the verbal abilities, due to the very simple questions, which do not require sophisticated language abilities. In kindergarten, children are exposed to verbal problems in different areas, in different interactions, and in different places; therefore, it is likely that both monolingual and bilingual children were exposed to verbal problems in simple language, acquired tools to deal with them, and consequently were able to solve verbal problems.

In conclusion, it can be seen that in most of the early numeracy literacy abilities, no differences were observed between the groups of monolingual and bilingual children, except for the numeric knowledge task, which requires linguistic knowledge, such as counting and naming. These findings reinforce the understanding that the linguistic knowledge of bilingual children should also be strengthened in the numeracy field. Also, it is very likely that upon arrival at school, formal learning will include many components of linguistic numeracy knowledge, and if language skills linked to numeracy are stronger, children will be better able to deal with more complex exercises that require verbal knowledge.

### The Relationship Between Linguistic and Numeric Abilities

The third question examined whether there is a relationship between the different early numeracy and literacy abilities among monolingual and bilingual children in the study and whether the relationships differ in the different groups. Correlations were found among most factors, but weaker connections were found among the bilingual children as compared to monolingual children, only between the orthographic knowledge and the linguistic knowledge and several numeric abilities. In general, the relationships found between the different abilities are consistent with the hypothesis that early exposure to language is important and influences the development of mathematical abilities (Vukovic and Lesaux, 2013), but the types and levels of relationships differ. In addition, the strong association between language abilities and numeracy abilities is manifested early in development and has been previously observed among preschoolers (Purpura and Reid, 2016). Furthermore, the current results are also consistent with findings from a previous study, which found that the link between language and mathematics was stronger among native speakers than among second language speakers (Peng et al., 2020).

In addition, a different pattern of connections was found between the two groups of children in the quantity comparison task. Among the monolingual children, no connection was found with any of the literacy tasks, but the bilingual children’s performance on this task was correlated with the phonological and orthographic factors. However, another study that examined the relationships between numeracy and verbal abilities among monolingual and bilingual children found no connections between the quantities comparison and the verbal tasks in either group of children (Bonifacci et al., 2016).

The most significant relationship was found between the simple calculation and number knowledge tasks and the three verbal factors in both groups of children. It seems that both counting and the process of calculating a simple sum involve literacy abilities in a significant manner. This finding can be strengthened by a study suggesting that basic mathematical skills, such as solving word problems, rely at least in part on verbal cognitive processing that may be difficult for those who have not yet mastered the language (Van Rinsveld et al., 2016). It is possible that the calculations are related to learning ability in general and not necessarily specifically to language.

These findings of the numeric knowledge factor are also consistent with our hypothesis. It is likely that if a numeracy task that involves linguistic knowledge of naming numbers and counting, a connection will be found with various verbal abilities. This is also the type of task in which the bilingual children performed worse than the monolingual children, which strengthens the involvement of linguistic abilities in this factor. Hence, when low abilities were observed in both literacy tasks and this type of numeracy task, it is likely that a strong association between these skills will be found.

In addition, in the verbal problems factor, a connection was found between phonological awareness tasks and verbal knowledge and vocabulary but not with orthographic knowledge among the bilingual children. This may be because understanding of the verbal story is required before you can perform the sum, and therefore, solving verbal problems is a cognitive task that relies heavily on language skills (Van Rinsveld et al., 2015).

Furthermore, the fact that comparing quantities was found to be connected with the phonological and orthographic factor is surprising and should be further investigated, in order to better understand this relationship. Previous studies found that the task of estimating quantities was not related to verbal abilities, but to intuition of numerical size, and it uses areas in the brain related to visual-spatial processing rather than language-related areas, which is contradiction to the current results (Dehaene et al., 1999). It may be that the visual scanning is required to perform this task and the orthographic tasks as well among the bilingual group.

It is important to note that only several of the correlations were found to be significantly higher among the monolingual children as compared to the bilingual children, and it was only between the linguistic and orthographic knowledge and numerical knowledge and the different calculation skills. No significant differences were found in the phonological factor and the comparison of quantities factor, and this may be because there was no difference between the two groups of children in these factors, and these abilities are more domain-specific and not connected to other factors, which share more abilities that are common (numeric and linguistic). These results strengthen the assumption that there is a strong connection between specific linguistic and numeric abilities and that the better performance of the monolingual children in the linguistic and orthographic knowledge strengthens their number knowledge and calculation abilities.

## Limitations and Conclusion

There are a number of limitations in the present study. First, we did not classify the bilingual group according to the level of knowledge of the Hebrew language, although for all children, the level of Hebrew was good enough to perform the tasks. It is possible that if the bilingual group had been divided according to the level of exposure to Hebrew at home, the results would have been expressed in a different way. In addition, the level of control of the home language was not tested either. Finally, the bilingual group was not divided according to the additional language they speak. It may be that the different languages affect different abilities based on their similarity to the other language. Therefore, it may important to examine the bilingual children in both languages they speak in future studies in order to investigate the differences in their performance in L1 and L2. This point is especially important for the number knowledge factor, while counting was taught in Hebrew only at the kindergarten to all children; the bilingual children might have been exposed to counting in another language at home. It is a very interesting point, and future studies should check the bilingual children in counting in both languages.

It is also important to continue to monitor children at different ages in order to see the differences after entering school and formal learning that can reduce or neutralize any negative impact of the second language. Perhaps as children get older, knowledge of another language can actually contribute to better performance, as found in other studies.

This study has educational implications for fieldwork. This research added to our knowledge about the numeracy and linguistic abilities of bilingual children, as well as the significant skills that are important to strengthen, expose, encourage, and improve during the day in kindergarten. It is critical to emphasize these skills in order to promote school readiness among this group. As noted in the literature, identifying a different profile of bilingual children from monolingual children may help in tailoring their learning to help them succeed and in creating learning goals unique to them, such as developing literacy in both languages (McCardle and Hoff, 2006).

According to the findings of this study, insufficient linguistic knowledge and vocabulary in the language spoken at school is one of the main difficulties of bilingual children. Hence, during the kindergarten period, it is important to work on language knowledge and vocabulary expansion among populations that speak more than one language in order to strengthen the language skills and abilities that affect additional skills.

## Data Availability Statement

The raw data supporting the conclusions of this article will be made available by the authors, without undue reservation.

## Ethics Statement

The studies involving human participants were reviewed and approved by University of Haifa, Faculty of Education Ethics Committee. Written informed consent to participate in this study was provided by the participants’ legal guardian/next of kin.

## Author Contributions

LB and SS conceptualized this study and contributed to the writing and interpretation of the data. LB contributed to data collection and wrote the first draft. SS did all the revision and performed the statistical analysis. All authors contributed to the article and approved the submitted version.

## Conflict of Interest

The authors declare that the research was conducted in the absence of any commercial or financial relationships that could be construed as a potential conflict of interest.

## Publisher’s Note

All claims expressed in this article are solely those of the authors and do not necessarily represent those of their affiliated organizations, or those of the publisher, the editors and the reviewers. Any product that may be evaluated in this article, or claim that may be made by its manufacturer, is not guaranteed or endorsed by the publisher.
